# TaiNi: Maximizing research output whilst improving animals’ welfare in neurophysiology experiments

**DOI:** 10.1038/s41598-017-08078-8

**Published:** 2017-08-14

**Authors:** Zhou Jiang, John R. Huxter, Stuart A. Bowyer, Anthony J. Blockeel, James Butler, Syed A. Imtiaz, Keith A. Wafford, Keith G. Phillips, Mark D. Tricklebank, Hugh M. Marston, Esther Rodriguez-Villegas

**Affiliations:** 1 Department of Electrical and Electronic Engineering, Imperial College London, London, UK; 2 Eli Lilly and Company Limited, Windlesham, UK; 3 Department of Neuroimaging Sciences, Institute of Psychiatry, Kings College London, London, UK; 4 TainiTec Ltd., Barking Road, London, UK

## Abstract

Understanding brain function at the cell and circuit level requires representation of neuronal activity through multiple recording sites and at high sampling rates. Traditional tethered recording systems restrict movement and limit the environments suitable for testing, while existing wireless technology is still too heavy for extended recording in mice. Here we tested TaiNi, a novel ultra-lightweight (<2 g) low power wireless system allowing 72-hours of recording from 16 channels sampled at ~19.5 KHz (9.7 KHz bandwidth). We captured local field potentials and action-potentials while mice engaged in unrestricted behaviour in a variety of environments and while performing tasks. Data was synchronized to behaviour with sub-second precision. Comparisons with a state-of-the-art wireless system demonstrated a significant improvement in behaviour owing to reduced weight. Parallel recordings with a tethered system revealed similar spike detection and clustering. TaiNi represents a significant advance in both animal welfare in electrophysiological experiments, and the scope for continuously recording large amounts of data from small animals.

## Introduction

Recent advances in the development of transgenic mice have provided unprecedented insight into the mechanisms of mammalian brain function and human disease processes, and have led to a dramatic shift from rats to mice as the preferred preclinical model used in drug discovery. However, their small size makes neuronal recording in freely-moving mice challenging. The ability to make direct electrophysiological recordings from populations of neurons requires multiple parallel recording channels and high sampling rates (>10 KHz) in order to properly characterize action potentials^1, 2^. The circuitry required is consequently energy-intensive and traditionally requires a multi-wire tether to provide power and to carry the analogue signal to the recording equipment. While this is practical in larger rodents, it presents a serious burden for a mouse^3^. Recently developed wireless recording systems allow both greater freedom of movement and the possibility of entirely new experimental designs, such as recording from complex enclosed environments^4–9^. However, there has always been a trade-off between the weight of the device and recording density or duration. Current off-the-shelf solutions are limited to less than 4 hours recording unless a harness is employed to support the additional battery weight, or provide longer recording only at reduced sample-rates which are suitable for recording local field potentials (LFP) or electroencephalograms (EEG), but not action potentials (APs). In either case, the devices are also still cumbersome and relatively heavy (4 g or more, Table 1). This represents >10% of the weight of an adult mouse - similar to a human subject carrying a 6 Kg weight on their head. To improve on existing wireless technology, the circuitry needed to be redesigned from the bottom-up, with an emphasis on improving the energy efficiency.

**Table 1:** A comparison of telemetry systems for electrophysiology.

System	Weight (g)	Time (hours)	Channels	Sample-rate (Hz)	Band-width (Hz)	Bit-depth	Noise (rms)	Input Voltage Range
TaiNi	1.5	72	16	19531	0.35–9700	12	3.9 µV	13 mVp-p
Millar MT10B^a^	2.5	N/A	2	2000	2–440	12	NA	2.5 mV
TBSI W16	4.0	4.2	16	30000	0.8–7000	16	8.3 µV	4 mVp-p
EMKA rodentPACK	5.2	150	4	1000	1–150	12	NA	±2 mV (EEG)
Multichannel systems W2100-HS16	6.8	1.7	16	25000	1–5000	16	1.9 µV	±12.4 mV
Neuralynx CUBE-64	17.0	0.5	64	30000	0.1–8000	16	2.5 µV	±5 mV
Pinnacle 8200-K6-SL^b^	23.0	36	3	2000	? (<1000)	12	NA	NA
Dueteron Technologies MouseLog-16^c^	2.8	9	16	29300	1–7000	NA	NA	10 mVp-p
TSE systems Neurologger^b,c^	2.5	92	4	500	? (<250)	NA	NA	NA

^a^Inductive charging system (no maximum recording time).

^b^Band-width was not specified in available materials, and is inferred.

^c^Data-logger systems (no transmission).

**Figure 1:**
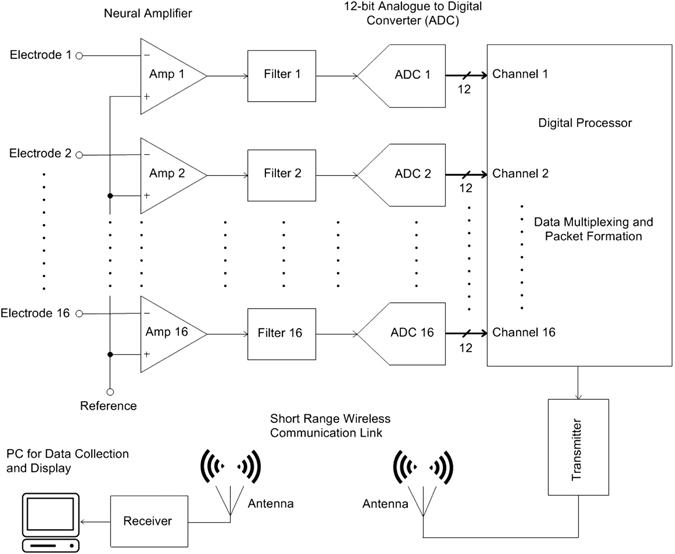
Circuit block diagram of the TaiNi system.

Here we test the performance of TaiNi - a novel wireless system based on a customized Application-Specific Integrated Circuit (ASIC) created using innovative low power design techniques. The circuit blocks, as shown in Fig. 1, incorporated in the ASIC include filters, amplifiers, analogue-to-digital converters, multiplexing and wireless transmission. This maximized efficiency and reduced total weight to 1.5 g. This system represents a significant advance in scalable recording systems for small rodents, and provides a robust foundation for additional designs.

## Methods

### Bench tests

#### Packet loss

Bench tests to assess the packet loss of the TaiNi devices were performed with the receiving antenna oriented horizontally (25 cm above the bench) and directly connected to a receiver (USRP X300, Ettus Research, USA) equipped with a pair of wideband transceiver daughter-boards (SBX-120 USRP). The TaiNi device was positioned on the bench, with the loop of the transmitting antenna directed towards the receiving antenna. The horizontal distance between the two antennas was increased in 10 cm increments between 0.1 and 3.0 m. With the transmitter operating at 2.31 GHz, the packet loss was quantified over a 2 minute period in each location.

#### Frequency response

The frequency response of the system was calculated using two series of chirp signals (arbitrary waveform generator 33522A, Agilent Technologies, USA), one low, and one high. For the low-frequency, a 2 mV peak-to-peak sinusoid with a logarithmic frequency sweep from 0.1 Hz–1 Hz was used. The sweep time was 100 s. For the high-frequency response we used a 10 s 2 mV 1–100,000 Hz logarithmic sweep.

### Animals

All experiments were performed in accordance with the United Kingdom Animal (Scientific Procedures) Act 1986 with approval from the United Kingdom Home Office.

For the current experiment 16 female rTg4510 mice^10, 11^ were bred by Envigo (Loughborough, GB), before being delivered to Eli Lilly and Company for testing (Windlesham, GB). Here, mice were singly housed on a 12 hour light/dark cycle with water provided ad libitum. Food was restricted as required prior to behavioural testing, with mice maintained at no less than 85% of their free-feeding weight. Because the mice used had previously participated in another experiment, 9 of them were treated from 2–3 months of age with doxycycline (DOX), initially via a daily bolus of 10 mg/kg p.o. doxycycline hyclate (Sigma Aldrich, GB) for the first two days. They were then maintained on doxycycline-mixed chow diet (Harlan Teklad Rodent Diet, 200 mg DOX per kg of dietary chow) until the end of the study. All tests involving a comparison of different systems required all animals to run in all conditions, so the DOX treatment could not affect the results.

### Surgery

Mice were implanted with either a single shank 16 channel probe attached to a microdrive (Cambridge Neurotech, GB) targeting the CA1 region of the hippocampus (n = 14 mice; AP: +1.7 mm, ML: −0.3 mm relative to Bregma), or a static dual shank silicon probe (n = 2 mice; 8 sites per shank (Neuronexus, USA)) targeting the medial prefrontal cortex (AP: +1.7 mm, ML: −0.3 mm relative to Bregma & DV: −2.0 mm from brain surface) & dorsal hippocampus (AP: −1.9 mm, ML: −2.0 mm relative to Bregma & DV: −2.0 mm from brain surface).

Mice were initially anaesthetised with 3% isoflurane, before being transferred to a stereotaxic frame and maintained for the remainder of the procedure at 1.5–2%. Five stainless steel screws (00–96 × 1/16, PlasticsOne, USA) were attached to the skull and used as an anchor point for implant. One of the screws was placed over the cerebellum and acted as the ground electrode. Craniotomies were made at the specified stereotaxic coordinates, and the probe lowered into position before the hole was sealed with silicone sealant (3–4680 silicone gel kit, Dow Corning, USA). Light curable composite (Revolution Formula 2, Kerr Dental, USA) was used to bind the implant/microdrive to the skull. Mice were allowed to recover for a minimum of 2 weeks before entering into any experiments, however probes attached to microdrives were slowly lowered to span the CA1 pyramidal layer over multiple days between 1 & 2 weeks post-surgery (initially implanted 500 µm below the brain surface).

### Electrophysiological recording

#### Room and system configuration

All *in vivo* tests were conducted in a 3.1 × 4.0 m windowless room with black walls and ceiling, lit by two uplighters and separated by a door from an anteroom containing the recording equipment. The room was on a 12-12 dark-light cycle with lights-on at 7am, and all experiments except the 72-hour recording were conducted in the light portion of the cycle. The room contained four bespoke infrared tables (100 × 100 cm), two on either side of the room and separated by 90 cm. A near- infrared emitter array (4 x IR emitters, ~650 nmm wavelength) and a miniature infrared-sensitive video camera (model M700LD, Henrys, UK) were centrally positioned above each table. The tables and emitter provided illumination of the subject from above and below to facilitate high-quality video tracking under a variety of lighting conditions.

To receive the signals from the TaiNi transmitter, each table was equipped with a pair of omnidirectional rod-antennas (CSL 12dBi, 2.4 GHz) elevated 30 cm above the table, oriented horizontally and at 90-degrees to one another, forming an open bracket around the recording environments described below. The two antennas from each of the two tables on either side of the room were connected to a 4-way splitter (model DBD-PD-8426-4, dBD Communications UK) by 1.5 m lengths of flexible antenna cable (LBC195 ExtraFlex, MS Distribution UK). Each splitter was in turn connected to one of two inputs on the receiver in the anteroom via 12 m lengths of heavy-gauge coaxial cable (LBC 400, MS Distribution UK). This heavy cable has very low 0.2 dB/m signal-attenuation.

In the anteroom, video signals from all four cameras were fed to a quad video combiner (VQM801P, Sanyo, JPN) and the merged video stream processed by EthoVision XT 8.5 (Noldus, NL), enabling the position of each mouse (body-centre and nose) to be tracked. To allow synchronization of the electrophysiology and behavioural data, an I/O interface (PTIO-0020, Noldus, NL) was used to generate a signal at the beginning and end of each video tracking recording, which was converted to a TTL pulse by a hardware interface (MED-PC model SG-233-48) and was recorded by the Taini acquisition PC. The synchronization was performed on the receiving unit during the data post-processing using the local timings of the TTL pulse recorded in both the electrophysiology and behavioural data. The antenna signals were fed to the same model and configuration receiver used in the bench tests (see above). Acquired signals were passed via a 10 gigabit ethernet cable to a Linux workstation running custom software for channelization, demultiplexing, visualization and storage. Channelization was parallelized using the graphics processing unit (Quadro K420, Nvidia) to handle the large data bandwidth. The software also registered TTL sync-pulses sent by the EthoVision PC.

All 16 channels from each transmitter were recorded “single-ended” (non-differential), using only the cerebellar skull screw as the signal ground.

#### Training and testing

During the 72 hour recordings, mice (n = 2) with dual shank probes were placed in a novel home-cage (15 × 30 cm) with free access to food and water for the duration of the recording. Open field pellet-chasing experiments were performed using 4 food restricted, five month old female rTg4510 mice (2 nonDOX & 2 DOX treated) implanted with single shank silicon probes. These mice were trained twice daily on a pellet chasing task (10 minutes per session) for a total of 10 days in the month proceeding testing. Training consisted of mice being placed in a transparent, circular arena (diameter: 30 cm; height: 50 cm) with pellets (12 mg chocolate flavour AIN-76A Rodent Tablet, TestDiet, USA) dispensed from above at 20 s intervals. On the day of recording, a 60 min sleep recording was performed in a familiar home-cage environment before the mice were transferred to the familiar open field arena for 20 mins of pellet chasing (20 s inter-pellet interval).

Throughout both the 72 hour and open field recordings, the cameras above each mouse continually captured data. In the open field recordings the animal’s body was tracked as a silhouette against the infrared emitting table under the arena, while during the home-cage recordings the overhead infrared emitters provided the necessary contrast against the bedding material, which obscured the light from the table below.

#### Split signal tests

Concurrent home-cage recordings (n = 3 mice implanted with single shank probes in CA1) were performed using the TaiNi and tethered Digital Lynx SX (Software: Cheetah v5.6.0, Neuralynx, USA) recording systems by utilising a signal splitter placed inline between the silicon probe and headstages. For the Neuralynx recordings, data was band-pass filtered at 0.5–9000 Hz, and sampled at 20 KHz.

### Weight tests on the automated T-maze

11 food restricted, five month old female rTg4510 mice (6 nonDOX & 5 DOX treated) were tested on a non-match to place T-maze task. The mice had previously been tested on the maze at 2 months of age and also in the week preceding this weight comparison test. On each of the 3 testing days, mice were randomly allocated a treatment condition (no transmitter, Triangle Biosystems International (TBSI) W16 transmitter or the TaiNi transmitter) in a fully counterbalanced manner.

The T-maze apparatus was custom-made (Apogee Engineering Analysis solutions, GB) and consisted of a matt black Perspex track (width: 6 cm) with clear Perspex walls (height: 20 cm). The centre-arm of the maze was 100 cm in length and each of the reward arms 36 cm long. The maze was raised 100 cm from the ground and sited in a rectangular room containing multiple visual cues. 3 pellet dispensers were situated around the maze (one per reward arm & one in the delay area at the beginning of the centre arm) to allow rewards (12 mg unflavoured AIN-76A Rodent Tablet, TestDiet, USA) to be delivered for successful performance.

The maze protocol was automatically controlled by a microcontroller board (Arduino Mega 2560) connected via a USB serial connection to a host-PC running Matlab (Mathworks, Natick, MA, USA). The animals’ position was detected by infrared beam breaks, which enabled the maze controller to adjust the position of 5 pneumatically driven, black Perspex doors. These could be to be raised from below to block entry into areas of the maze as appropriate.

The T-maze task comprised of a sample and choice component. In the initial forced phase, mice were guided along the centre arm by the doors and forced to turn left/right in a pseudo-random manner (maximum of 3 consecutive sample runs to the same arm) in order to receive a reward. Mice then returned to the holding area in return for a second reward, at which time the choice phase of the task began. Mice were again guided along the central arm of the maze, before being offered a free choice to enter either reward arm. Mice were given a reward only if they chose to enter the opposite arm visited during the sample phase. A further reward was provided at the waiting area for mice making a correct choice. Mice were held in this zone for 5 s prior to be start of the next trial. On each day mice were given 60 minutes to complete as many trials as possible.

### Statistics and Analyses

Running-speed estimates were calculated from the animals’ body centre location (x/y coordinates) integrated over 400 ms non-overlapping epochs^12^. Nose-position was used to provide the high-precision data required for analysing neuronal spatial firing properties. Immobility was defined as a period when the mouse moved at a rate of less than 0.5 cm/s for at least 300 s. Running was defined as any period where the mouse moved at more than 5 cm/s for at least 100 ms. For the automated T-maze weight tests, a repeated-measures ANOVA was used, with orthogonal planned contrasts between the NONE and TaiNi condition, and between TaiNi and TBSI.

Local field potentials (LFPs) were analysed using an anti-aliased version of the raw data, downsampled to 1 KHz. A short-time FFT using 2-second sliding windows with 50% overlap was used to estimate spectral power. Ripple-detection was performed according to the methods described by Sullivan *et al*.^13^. Phase-amplitude-coupling in the hippocampus was analysed according to the “cross-frequency-coupling” methods described in Onslow *et al*.^14^ A simple Pearson’s correlation was used to analyse the relationship between 0.5–4 HZ delta power and ripple density.

Spike detection and cluster analysis was performed using the KlustaKiwk suite of software^15^, with the maximum number of clusters set to 100 and using the Akaike Information Critierion^16^ to determine whether a given cluster should be divided. For spike-detection, the data was filtered (500 Hz high-pass 3rd-order Butterworth filter) and z-scored, with a 4.5 standard-deviation detection threshold. Clusters were discarded if their autocorrelograms exhibited excessive spiking in the 2 ms refractory zone, and were combined where they shared clear refractoriness with other clusters. Place fields for each cluster (putative neuron) were generated according to the methods described previously^12^, but using a linear colour scale for representing firing rates. Briefly, mean dwell-time (seconds) and spike-counts in 1 × 1 cm bins were calculated to generate the firing rate in each bin, before applying a 5 cm Gaussian smoother to the map.

### Data Availability

The data that support the findings of this study are available from the corresponding author upon reasonable request.

## Results

### System overview and bench-tests

**Figure 2:**
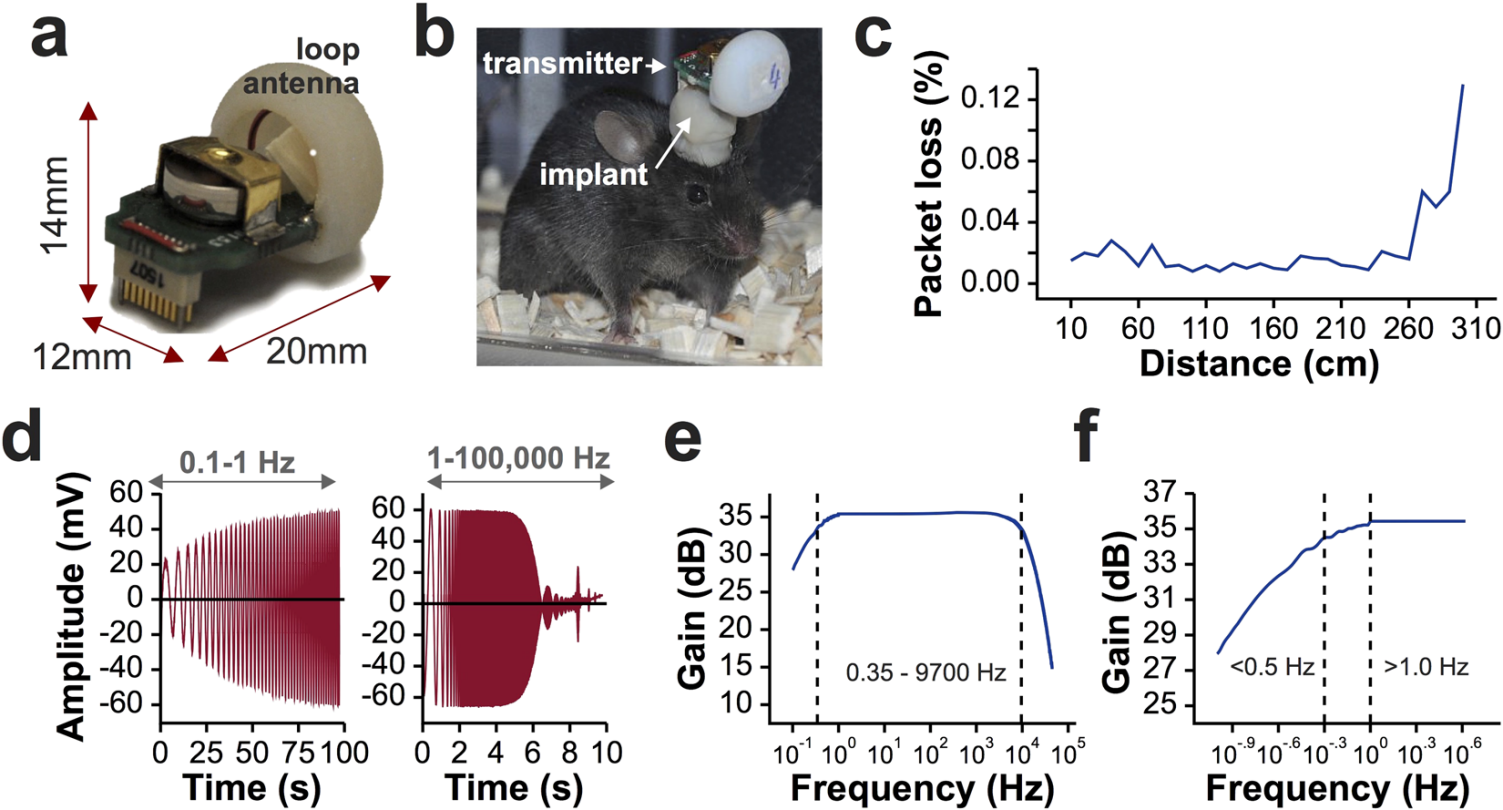
The TaiNi transmitter. (**a**) The device with battery and antenna-housing in place. (**b**) In use with a mouse. Orientation of the device depends on the orientation of the implant on the mouse’s head. (**c**) Packet loss using a single antenna elevated 25 cm above the bench on which the transmitter was positioned. (**d**) Time-domain output from the system, given a constant amplitude +/−2 mV input with frequency sweep from 0.1 Hz to 100 kHz. (**e**) Log-scale frequency response. (**f**) Enlarged view of the lower end of the frequency response, showing slight signal attenuation in the slow-wave/lower-delta frequency range.

The TaiNi system (Fig. 2a) weighs 1.5 g (including battery) and transmits 16 channels sampled at 19.531 KHz. The system employs a high-pass hardware filter (−3 dB at 0.35 Hz) to reduce high-amplitude, low-frequency signal components, yielding a total bandwidth (after anti-aliasing) of 9.7 KHz. The device measures 20 × 12 × 14 mm and includes a plastic shield for the loop antenna. Omnetics connectors were used for the current version of the system, although they could easily be changed without altering performance. A summary of the system’s electrical specification is given in Table 2. Figure 2b shows the device in use, with the transmitter comprising about 50% of the volume of materials on the mouse’s head, the rest being the electrode implant assembly as described below. For bench tests, data from a single transmitter was detected by an off-the shelf 2.4 GHz WiFi antenna elevated 25 cm above the bench. Signals were fed to a receiver (Ettus, model USRP X300) and passed via a gigabit ethernet link to a Linux (Ubuntu) workstation running custom acquisition software. For all recordings, we used a 180 mAh zinc-air battery weighing 0.58 g.

**Table 2:** A summary of the TaiNi system’s performance.

Feature	System Performance
Supply Voltage Range	1–1.4 V
Average Current Consumption	2 mA
Peak Current Consumption	2.5 mA
No. of Channels	16 channels
Bandwidth	0.35 Hz–9.7 kHz
Number of Bits	12-bit
CMRR (Common Mode Rejection Ratio)	78 dB
PSRR (Power Supply Rejection Ratio)	>46 dB
Input Referred Noise	3.9 µV
Transmission Range	2.5 m (Received SNR = 20 dB)
Transmission Protocol	Customized protocol with packet size of 810 bits
Battery Lifetime	Over 72 hours
Transmission Modulation	BASK
No. of Devices for Simultaneous Recording	4
Ground Connector Impedance	0.2 Ω

Packet loss was averaged over two minutes at each distance from 10 cm to 300 cm in 10 cm steps (Fig. 2c). Average loss was 0.015% (1.5 packets per million) for distances under 260 cm, rising to 0.13% at 300 cm. The frequency response of the device was estimated using a 100 s chirp signal ranging from 0.1 Hz to 1 Hz, and a 10 s chirp ranging from 1 Hz to 100 KHz (Fig. 2d). There was no signal attenuation between 1 Hz and 2000 Hz (Fig. 2e). The 0.5 Hz signal was only attenuated by 0.95 dB at 0.5 Hz, below the generally-held lower-bound for delta oscillations^17^, and by 7.5 dB at 0.1 Hz (Fig. 2f).

### *In vivo* tests

Female Tg4510 mice (fourteen 5 month olds and two 9.5 months old) which had been used in previous experiments were used to test the impact of the TaiNi system on behaviour and its performance in experimental settings. Each mouse was implanted with a 16-channel silicon probe (Cambridge NeuroTech, UK) directed at the dorsal CA1 region of the hippocampus. For the younger mice, the electrode was fixed to a miniature microdrive to allow positioning of the recording sites in the pyramidal cell layer.

#### Behavioural impact: TaiNi versus existing wireless technology

Supplementary video 1 shows a mouse wearing the TaiNi system and exhibiting typical head-bobbing movements, as well as very normal looking exploratory behaviour, rearing and grooming. The same mouse was recorded 10 minutes later in Supplementary video 2, wearing a lightweight 16-channel head-stage and recording tether attached to a load-bearing cable-management system.

**Figure 3:**
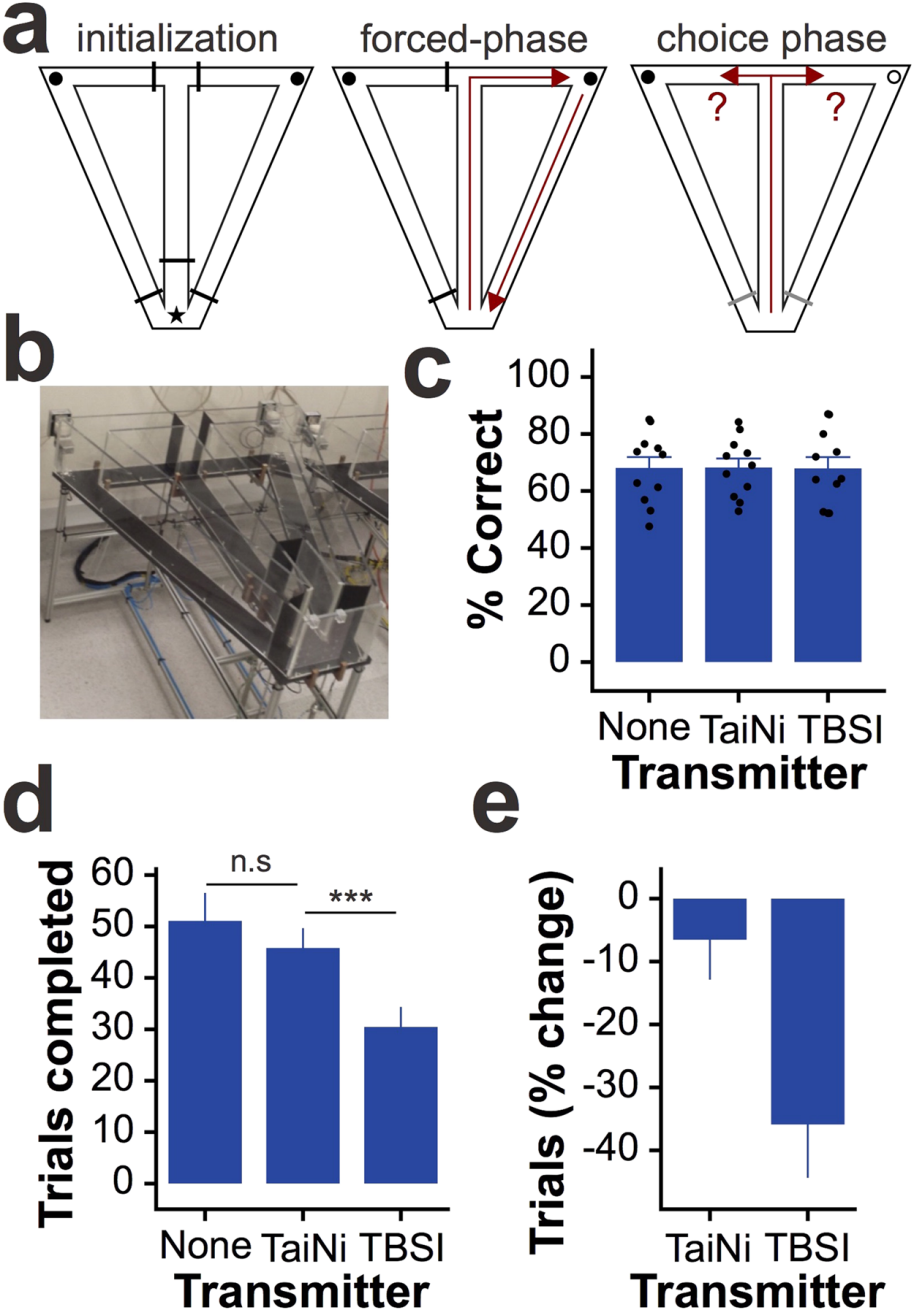
Behavioural impact of the TaiNi transmitter. The TaiNi transmitter. (**a**) The design of the automated T-maze task. Left: all doors (black bars) are raised and the mouse is confined to the waiting-zone (star). Both reward locations (circles) are baited with food. Middle: only one door is opened, forcing the mouse to retrieve one of the rewards and return to the waiting-zone. Right: both doors at the choice-point are opened and the mouse must remember which of the two rewards still remains. (**b**) A photo of one of the mazes. (**c**) The percentage of correct choices under each transmitter condition (NONE = no transmitter). Each mouse was run under all three conditions on three separate days. Dots show the data-points from each mouse. (**d**) The number of trials completed under each condition. (**e**) The percentage change in the number of trials completed, per-mouse, when wearing either the TaiNi or the TBSI transmitter, compared with the NONE condition. The TBSI transmitter induced a 35.9% reduction in trials completed. Error bars show SEM. ***p < 0.005.

To quantify the behavioural benefits of using an ultra-light system, we tested spatial working memory in eleven mice on an automated delayed non-match-to-place T-maze task (Fig. 3a,b). All animals had been previously trained to perform the task with no transmitter. On testing days animals were run in the maze wearing either no transmitter (NONE), the 1.5 g TaiNi, or a comparable 4.0 g commercially available transmitter (TBSI model W16). Over three days the mice were exposed to all three conditions in counterbalanced order. Figure 3c shows there was no difference in the percentage of correct choices from each animal under each condition. In contrast, mice completed significantly more trials with TaiNi compared with TBSI (repeated-measures ANOVA, n = 11, t = 4.29, p < 0.01), and there was no significant difference between TaiNi and the NONE condition (n = 11, t = 1.27, p = 0.23) (Fig. 3d). The magnitude of the impact of wearing the heavier TBSI transmitter is clear from Fig. 3e: 35.9% fewer trials than when tested wearing no transmitter at all, as compared with a 6.5% reduction in the TaiNi condition. Anecdotally, by the end of each session mice wearing the TaiNi transmitter were behaving normally, while animals wearing the heavier 4 g transmitter were clearly struggling to maintain an upright head posture.

#### Simultaneous high-density recording from 4 animals

**Figure 4:**
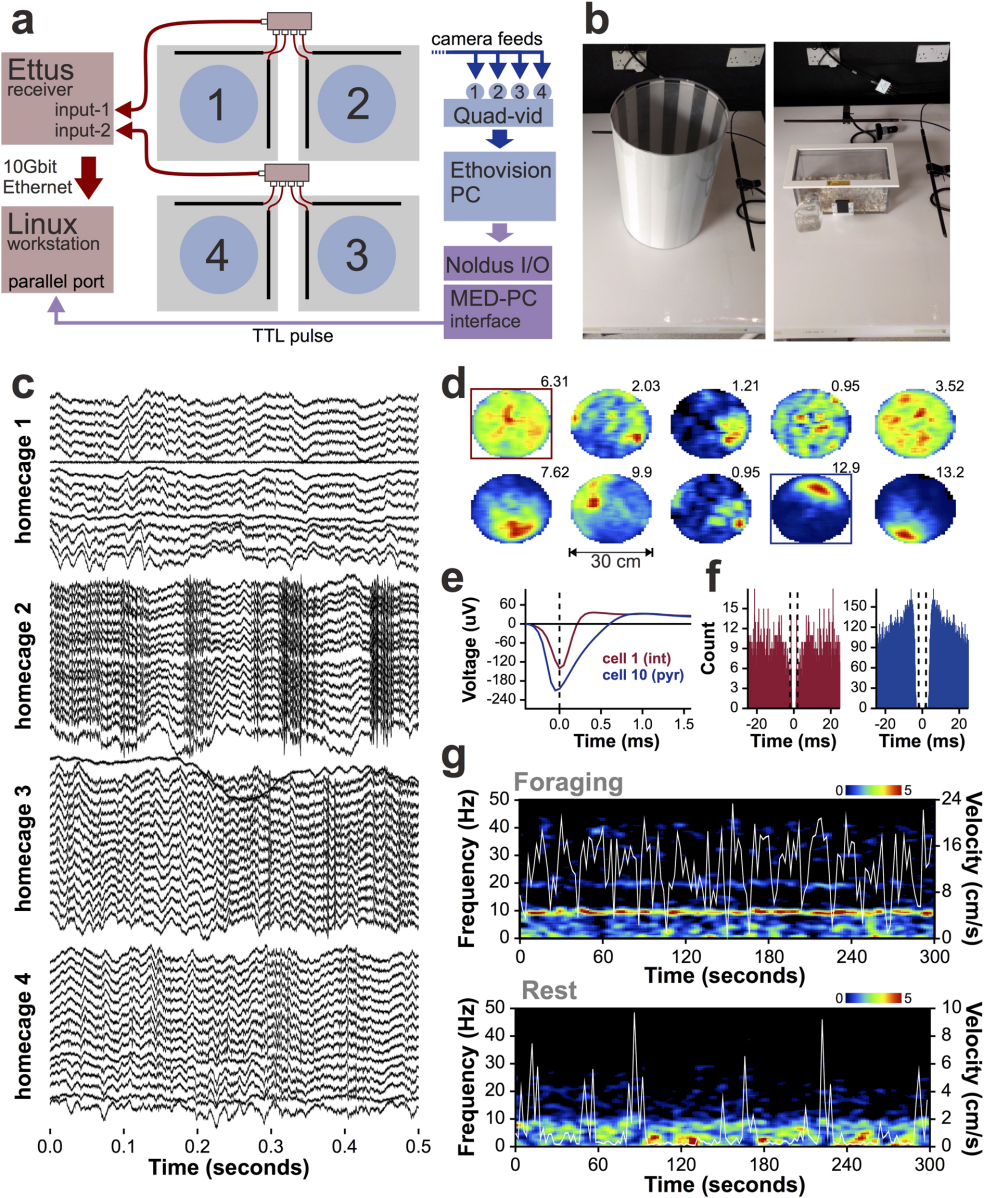
*In vivo* recordings with TaiNi. (**a**) Recording configuration diagram, illustrating integration of electrophysiological (pink/red) and behavioural (blue) data streams. Boxes in purple represent the sync-pulse pathway connecting the two systems. A separate Linux workstation and Windows PC run the TaiNi and EthoVision acquisition software, respectively. Boxes in purple represent the sync-pulse pathway connecting the two systems. Blue circles represent the pellet foraging arenas, Grey squares are the infrared tables. Black rods represent the antennas on each table, which feed to a 4-way signal-joiner. (**b**) The pellet-foraging arena (left) and the home-cage used for sleep/rest recordings (right). (**c**) Simultaneous recordings from 4 mice in the home-cages. Each mouse contributes 16 traces - one from each channel on the electrode. Local field potentials are evident in all animals. The mouse in home-cage 2 shows recordings indicative of muscular artefacts typical of chewing or scratching. Action potentials can be seen in the recordings from home-cage 4. (**d**) Spatial firing fields of 10 neurons recorded from the CA1 field of the dorsal hippocampus of one of the mice, during the pellet foraging task. Mean firing rates are indicated above each heat-map. Warm colours represent higher firing rates. The blue box highlights a putative pyramidal cell (place-cell) with a characteristic well defined firing field on the north side of the arena. The red box highlights a putative interneuron with a more diffuse firing pattern. (**e**) Mean action-potential waveforms of the interneuron (int) and pyramidal cell (pyr). (**f**) For the same two cells, the action-potential autocorrelogram, highlighting the wider refractory-period and burst-firing tendency (pronounced peaks at ±5 ms) for the pyramidal cell (blue). (**g**) Five minutes of the hippocampal power spectrum from a mouse chasing pellets in the arena (top) or resting in the home-cage (bottom). Running speed (cm/s) is overlaid in white. Note the frequent pauses during foraging which accompany momentary reductions in the 4–12 Hz theta band. During rest, brief bursts of movement interrupt the 0.5–4 Hz delta power.

We recorded LFP and action potentials from four animals on a backlit infrared table, monitored by overhead cameras connected to a PC running whole-body tracking software. Output of the TaiNi transmitter was picked up by two antennas per table, and fed to a single receiver. A schematic of the recording configuration is shown in Fig. 4a. A 30 cm diameter striped arena and a modified home-cage were used to record during alternating pellet-foraging and sleep/rest trials, respectively (Fig. 4b). The TaiNi transmitter and recording system comfortably handled 64 channels of data sampled at 19.5 KHz (16 channels per mouse). As illustrated in Fig. 4c, traces from each animal exhibited normal local field potential oscillations, evidence of action potentials, and in one of the examples shown, the muscular artefacts normally associated with chewing in a pellet-foraging task. A small number of defective channels were identified, but these were specific to each mouse and therefore represent a problem with the electrodes, not the TaiNi system. It is also worth noting that we observed some instances where a drop of water could disrupt performance, which was resolved by using an air-duster and absorbent cotton to remove the moisture. This will be addressed in the production version of the device by the addition of an extended housing to shield the battery enclosure and circuit board in a manner that does not impact the total weight.

Using the KlustaKwik and KlustaViewa offline spike sorting tools^15^, we detected action potentials and assigned them to individual hippocampal neurons. We combined the action potential data with the time-stamped whole-body tracking coordinates corresponding with the concurrent position of the animal’s nose. The spatial firing probability of each neuron was calculated as described previously^18^. Figure 4d shows the firing fields from one of the four mice. From this recording it is clear that we obtained a collection of pyramidal cells with well-defined spatial firing fields (place cells^19^), interneurons with more uniform patterns of activity^20^, and sparsely firing neurons which were essentially silent for most of the recording. Figure 4e compares the average waveforms of the putative pyramidal cell and interneuron highlighted in the previous panel. The spike-timing autocorrelograms for these two neurons (Fig. 4f), reveal the interneuron’s characteristic tonic firing probability, while the pyramidal cell tends to fire bursts of action potentials within 10 ms of one another^21, 22^.

Figure 4g shows a spectral analysis of the data from a single channel in the hippocampal pyramidal cell layer of the same mouse, comparing a foraging and a rest trial. The running-speed of the mouse is overlaid to highlight the high degree of temporal dependence of 0.5–4 Hz delta and 4–12 Hz theta oscillations on momentary running speed. During the foraging trial (top panel) the animal frequently pauses to wait for the next pellet to drop to the floor of the arena before resuming foraging. During these pauses we observed brief pronounced reductions in theta power, as expected. Conversely, during the rest trials, power in the 0.5–4 Hz delta band predominated, but was seen to be disrupted by momentary periods of arousal when the mouse began moving again.

The neuronal and LFP results described here would be impossible without confidence in the sub-second synchronization of the electrophysiology and behavioural data, indicating that the time-stamping for both the TaiNi and the EthoVision systems is very robust.

#### Battery life test: 72-hour recording

To test the battery-life of the device, we recorded behaviour and LFPs from two 9.5 month old Tg4510 mice during 72-hours of natural behaviour in a modified home-cage with *ad libitum* access to food and water. A small drop of hot-glue applied to either side of the connector ensured that the device remained attached for the duration of the recording. A small amount of electrical tape was used to shield the circuit board and battery enclosure from water. The home cages were placed centrally on an infra-red table and the room was placed on a 12-12 hour light cycle with lights-on at 7AM. During these 72 hours the animals were monitored via the overhead camera but nobody entered the room. Recording duration exceeded our expectations, with the devices continuing to function beyond the end of the 72-hour trial.

**Figure 5:**
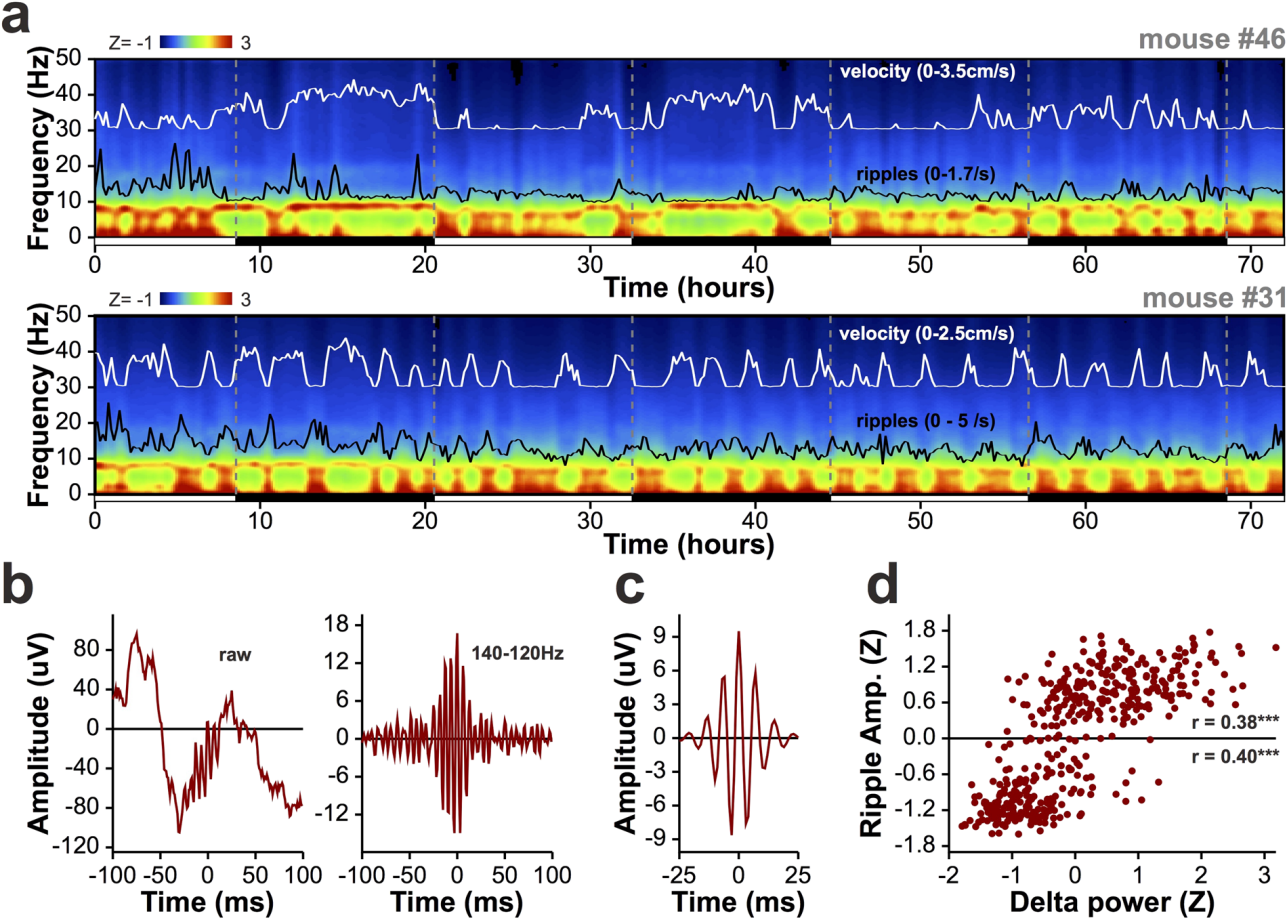
72-hour recordings from two mice in the home-cage. (**a**) The time-course of spectral power in the hippocampus across the 0–50 Hz frequency range for two mice. White lines show running speed, and black lines represent the density of 140–220 Hz ripple-oscillations, integrated in 10-minute bins. Black and white bars below each graph indicate whether the lights were on (white) or off (black) during the recording, and vertical dashed grey lines mark the transitions between light-cycles. The top panel represents a mouse with a clear diurnal pattern of activity, while the mouse in the lower panel has a much more erratic pattern of activity. For both animals, ripple density tracks power in the delta band, while theta dominates during periods of high mobility. (**b**) A representative ripple from mouse #31, before (left) and after(right) filtering in the ripple band. (**c**) The peak-aligned average ripple during periods of immobility from the 72-hour recording. This represents 16315 ripples collected during 1014 minutes. (**d**) For 10-minute non-overlapping bins spanning the entire 72-hours, mean ripple amplitude as a function of mean 0.5–4 Hz delta power in each bin. Both measures are normalized to the mean and standard deviation of the values across bins. The individual ripple-amplitude distributions above and below the mean are significantly, positively correlated with delta power. ***p < 0.001.

The spectral time-course of hippocampal LFP oscillations over 72 hours revealed slow cyclic variations in the balance between low- and high-frequency oscillations which followed the light cycles in one of the mice but not in the other (Fig. 5a). This difference is recapitulated in the time-course of running speed for the animals, with the second animal exhibiting long periods of activity in what is normally considered the rodent sleep-phase. This highlights the traditional difficulty in quantifying neuronal activity from a given sleep/wake state when experimenters are limited to shorter recordings. At this timescale it is evident that running and 4–12 Hz theta are associated with each other as well as with increases in the 30–100 Hz gamma power band and reductions in 13–30 Hz beta. The reverse holds during immobile periods, which exhibit low theta and stronger 0.5–4 Hz delta.

Analysis of the density of 140–220 Hz ripple oscillations (black trace, Fig. 5a), which are associated with slow-wave sleep and memory consolidation^23–25^, confirmed that their numbers increase during periods when delta power is highest. Figure 5b shows an unfiltered ripple from mouse #31, and the same ripple band-pass filtered at 140–220 Hz. Figure 5c shows the average filtered ripple for this mouse, aligned to the highest amplitude peak in each oscillatory event. We used 10-minute bins to compare the mean power in the 0.5–4.0 Hz delta band and the mean amplitude of detected ripple oscillations over 72 hours (Fig. 4d). For the 4320 10-minute epochs analysed, this revealed a robust Pearson’s correlation (r = 0.49, p < 0.001), and a clear bimodal distribution in ripple amplitude. This bimodality emerged only when the data was averaged on behavioural time-scales, capturing amplitude changes as the mouse transitions between low and high delta power states during running and immobility, respectively. However, using a z-score of zero as the arbitrary threshold for categorization, ripple amplitude was also strongly correlated with delta power for both low (r = 0.48, p < 0.001) and high (r = 0.38, p < 0.001) amplitude ripples.

#### Spike and LFP signal quality compared with 16-bit tethered system

Simultaneous recordings from TaiNi and the TBSI devices were impossible due to the size and shape of the respective systems, and the limited space on the mouse’s head. However we were able to create a signal splitter which allowed comparison of data acquired with 12-bit TaiNi to the same data processed via a tethered 16-bit system (Digital Lynx SX, Neuralynx). This is not an entirely fair comparison, given that tethered systems always offer greater bit-depth and/or voltage range to represent their input, compared with wireless devices. Nevertheless, we collected 10 minutes worth of recording from each of three mice, splitting the signal at the implant connector. We were particularly interested in whether the traces, power spectra, and measures of frequency coupling based on the LFP would be comparable. We also wanted to investigate how spike-sorting algorithms would handle the two datasets.

**Figure 6:**
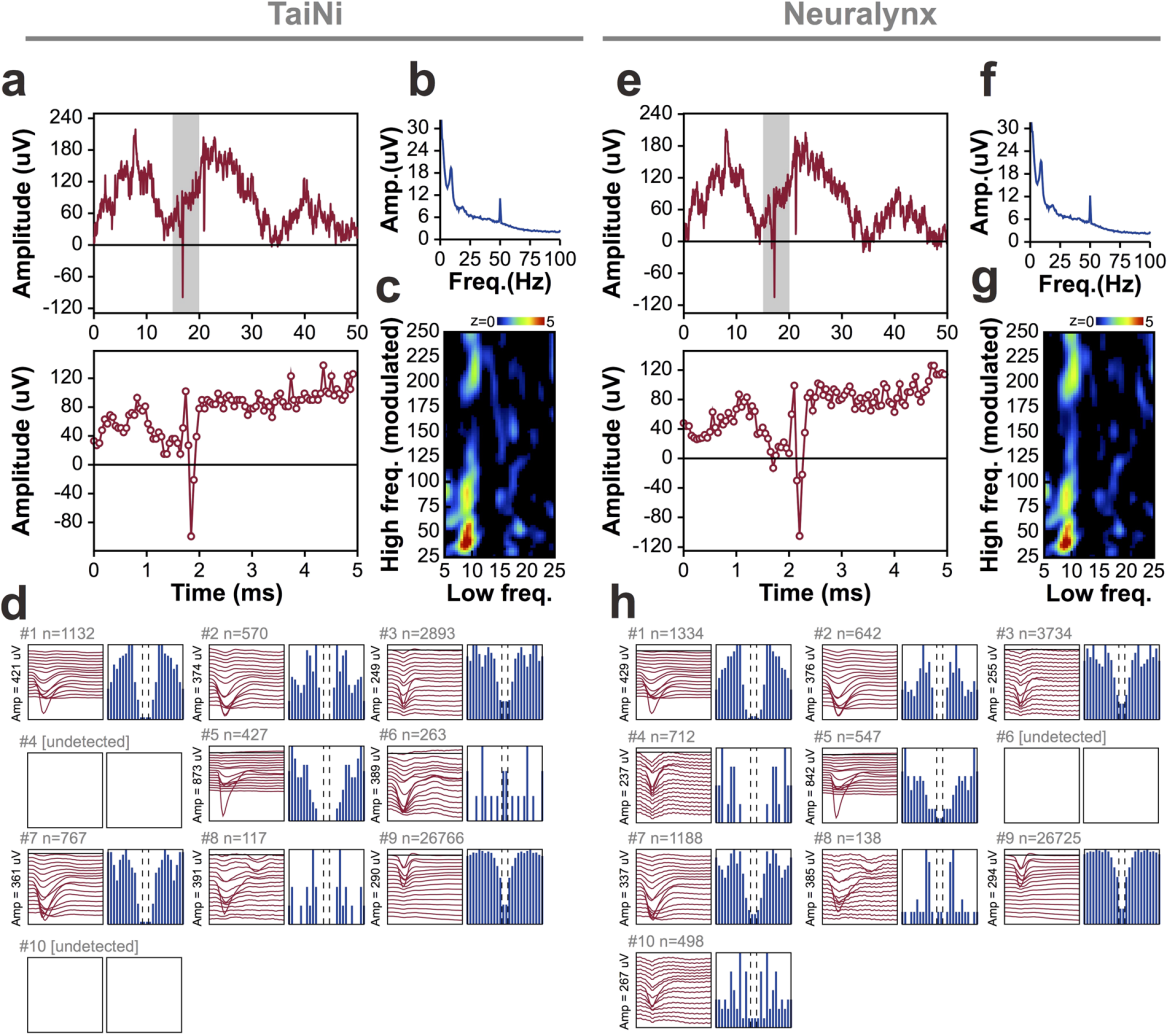
Comparison between the wireless TaiNi system (left) and a Digital Neuralynx tethered system (right). Data was split at the connector on the mouse’s head and fed to both systems simultaneously for comparison. (**a**,**e**) Matching raw traces as processed by the two systems, highlighting action potentials and theta oscillations. Bottom panels are enlarged views of the grey zone in the top panel. (**b**,**f**) Power spectra for the entire 10-minute recording. (**c**,**g**) Phae-amplitude coupling analysis, showing clear modulation of the amplitude of gamma (centred at ~37 Hz) by the phase of theta (centred at ~8 Hz) oscillations. (**d**,**h**) Comparison of spike-sorting results from the two systems, with identified cells (clusters) represented by their mean action-potential depth-profile (red traces) and their autocorrelograms (in blue). The display topographical with the same putative cell occupying the same position in (**d**) and (**h**). TaiNi tends to detect somewhat fewer spikes overall, and both systems sometimes identify clusters not found by the other. Overall however, the results are quite similar.

Figure 6 compares results from the two data streams, with TaiNi on the left and Neuralynx on the right. Sample traces from the CA1 pyramidal cell layer of the hippocampus in one of the mice are shown in Fig. 6a,e. The LFP appears virtually identical in both systems (top panel) while very subtle differences became apparent when looking at the data on a much shorter 5 ms timescale (bottom panel). However, the action potential in this example is extremely similar. Next we analysed the power spectrum using a short-time Fourier transform with a 1-second sliding window (Fig. 6b,f). The resulting spectra were also nearly identical. Next we looked at phase-amplitude coupling - a measure of the degree to which the amplitude of fast oscillations is modulated by the phase of slower ones. Both systems produced a robust peak in modulation of 35 Hz gamma by 9 Hz theta oscillations, as expected, with only minor differences in the pattern of background modulation (Fig. 6c,g). Finally, we tested whether the subtle differences in the signals would affect the classification of action potentials based on their waveforms across the 16 recording sites of the electrode. The depth-profile of the mean action potential waveform is shown alongside the 100 ms activity histogram for each detected cell (Fig. 6d,h **)**. A total of ten neurons were detected, 8 by the TaiNi device, and 9 by the Neuralynx system. Seven cells were detected by both systems, there was 1 cell detected by TaiNi only, and another two which were detected only by Neuralynx. For the seven cells detected by both systems, TaiNi detected on average 17.3 ± 4.1% fewer action potentials than Neuralynx. However, the cells identified by TaiNi had 55.9 ± 22.3% better refractoriness scores, suggesting that the action potentials excluded by TaiNi were lower amplitude and therefore more likely to be of ambiguous classification. It is worth noting that the sample size (7 common cells) is small, and running the same data through KlustaKwik does not yield identical results every time. Nevertheless we believe these findings, with this dataset, are representative.

## Discussion

Our tests confirm that the TaiNi wireless system is lighter than any commercially available wireless device able to record neuronal action potentials, and indeed lighter even than multi-channel devices capable only of recording LFPs. The light weight resulted in significant improvements in the ability to complete trials on the T-maze task compared to the most similar commercially available alternative, the TBSI transmitter. While our relatively unimpaired animals showed no concomitant reduction in the percentage of correct choices on the T-maze task, the ability to complete additional trials increases statistical power and reduces the number of animals required for a given experiment. This provides an animal welfare benefit beyond the obvious ones for the individual animal wearing the transmitter.

TaiNi was capable of undisturbed recordings spanning multiple diurnal cycles. This is particularly important for studies using mice, for which human contact has a prolonged effect on behaviour, and which often have (as we observed) erratic sleep patterns. Consequently the TaiNi transmitter both removes the stress associated with repeated handling to change batteries or untwist tethers, and also enables state-dependent analyses of neuronal activity which are traditionally very challenging in mice. Long-duration recording also enables the discovery of novel phenomena such as the discrete categories of 140–220 Hz ripple-oscillations revealed by the current experiments. This might not have been possible without many hours of recordings integrated across fluctuating behavioural states. While we were unable to process the enormous volume of 72-hour action potential data at the time of writing, the potential to do so in the near future opens up many novel avenues of scientific investigation. It is also worth noting that while our tests ran to 72 hours using a 180 mAh battery, using a 310 mAh better would extend recording to 120 hours (5 days) while adding only 0.25 g to the total weight.

We also confirm that the acquisition system is capable of simultaneously acquiring 16 channels of 19.5 KHz data streams from four devices using a single receiver, and accurately synchronising the data with movement and position information recorded by the Ethovision whole-body tracking software. This demonstrates the scalability of the system given additional receivers, and the time-stamp integrity required for analyses such as the spatial selectivity of hippocampal neurons and the instantaneous behavioural correlates of local field potential oscillations. Both benefits are important in industry-scale experimental programs where recording from many animals simultaneously is a benefit, and integration of multiple data streams is essential.

Finally, split-signal recordings confirm that, for LFP recordings, the results from TaiNi are qualitatively and quantitatively similar to those obtained using a higher-power, higher bit-rate tethered system. As expected, there is some compromise in terms of detection of very low amplitude events, which manifests as slightly different spike clustering results and somewhat lower action-potential counts in each cluster. However, we believe this is typical of any 12-bit wireless recording system, and does not represent an obstacle to collecting high-quality data.

In summary, we believe the TaiNi wireless transmitter is a transformative technology which opens up entirely new areas of investigation using long-duration, high-density recordings in mice. The experiments described here illustrate, for the first time, the capabilities of this device in a real-world experimental setting, and reveal a feature of high-frequency neuronal oscillations which might otherwise have been impossible to observe.

## Electronic supplementary material

Supplementary Video 1 - TaiNi

Supplementary Video 2 - Tether
